# Assessment of a Calibration-Free Method of Cuffless Blood Pressure Measurement: A Pilot Study

**DOI:** 10.1109/JTEHM.2022.3209754

**Published:** 2022-09-26

**Authors:** Cheng-Yan Guo, Chia-Chi Chang, Kuan-Jen Wang, Tung-Li Hsieh

**Affiliations:** Accurate Meditech Inc. New Taipei City 241406 Taiwan; Advanced Micro Devices Inc. Hsinchu 241046 Taiwan; Department of Electronic EngineeringNational Kaohsiung University of Science and Technology, Sanmin 517768 Kaohsiung City 807618 Taiwan

**Keywords:** Moens-Korteweg equation, blood pressure, mean artery pressure, pulse wave velocity, blood pressure monitor

## Abstract

This study proposes a low-cost, high-sensitivity sensor of beat-to-beat local pulse wave velocity (PWV), to be used in a cuffless blood pressure monitor (BPM). Objective: We design an adaptive algorithm to detect the feature of the pulse wave, making it possible for two sensors to measure the local PWV in the radial artery at a short distance. Unlike the cuffless BPM that needs to use a regression model for calibration. Method: We encapsulate the piezoelectric sensor material in a cavity and design an analog front-end circuit. This study used color ultrasound imaging equipment to measure radial arterial parameters, including the diameter and wall thickness, to aid the estimation of blood pressure (BP) using the Moens-Korteweg (MK) equation of hemodynamics. Results: We compared the blood pressure estimated by the MK equation with the reference BP measured using an aneroid sphygmomanometer in a test group of 32 people, resulting in a mean difference of systolic BP of −0.63 mmHg, and a standard deviation of ±5.14 mmHg, a mean difference of mean arterial pressure (MAP) of 0.97 mmHg, with a standard deviation of ±3.54 mmHg, and a mean difference of diastolic BP of −1.14 mmHg, with a standard deviation of ±4.08 mmHg. This study has verified its compliance with ISO 81060-2. Conclusions: A new type of wearable continuous calibration-free BPM can replace the situation that requires the use of traditional ambulatory BPM and reduce patient discomfort. Clinical impact: In this study can provide long-term continuous blood pressure monitoring in the hospital.

## I. Introduction

In recent years, wearable devices have begun to integrate a large number of functions into a system [1], and now they have begun to have health care and vital sign monitoring, such as pulse rate, blood pressure, oxygen situation, and arrhythmia detection [2]. Blood pressure is one of the vital signs of the human body [3], and it is also one of the research focuses of wearable devices with vital sign monitoring. The electronic blood pressure monitor (BPM) uses a cuff with the oscillometric method to measure blood pressure [4]. A cuff that can be inflated and pressurized to connect to the Wheatstone bridge sensor [5], and use the motor to increase the internal pressure of the cuff dynamically, make the cuff press the radial artery or brachial artery of the arm. When the internal cuff pressure and the arterial pressure are equal, the amplitude of the air oscillating inside the cuff will be the maximum. At this time, the cuff’s pressure is the mean arterial pressure (MAP) [6]. When the MAP position of the envelope of an oscillating signal of the cuff is determined, the systolic blood pressure (SBP) and diastolic blood pressure (DBP) can be estimated from the peak relative to the MAP position [7].

However, the oscillometric method must use components such as a cuff, motor, and valve to make the size of the BPM larger than wearable devices. On the other hand, in the traditional hypertension clinic monitoring scenario, non-invasive ambulatory blood pressure monitoring (ABPM) is a valuable tool for monitoring BP abnormality in the clinical setting. Monitoring a patient’s 24-hour blood pressure changes allows patients to manage their treatment better [8]. However, ABPM requires intermittent inflation of the cuff, which interferes with the patient’s daily life, causes discomfort, and reduces treatment compliance with blood pressure monitoring [9]. Cuffless BP monitoring technology can overcome patient discomfort when measuring for a long time. In order to enable wearable devices to achieve cuffless BP measurement, the current primary research is based on hemodynamics and measuring pulse wave velocity (PWV) to estimate artery pressure. According to the definition of a cuffless BPM in the Institute of Electrical and Electronics Engineers (IEEE) standard, BPM without an occluding cuff is defined as cuffless BPM [10]. An advantage of cuffless BPM compared to traditional BPM is that it enables a more comfortable and long-term BP monitoring to be realized [11]. The techniques used in this field include ECG combined with PPG, phonocardiographic sensors, elastic modulus, dimensions and stiffness of the intervening vessels, and other methods to measure physiological parameters. The selected technique builds a mathematical model to estimate BP. Commercial products such as Maisense’s Freescan use Electrocardiography (ECG) and Photoplethysmography (PPG) to measure pulse arrival time (PAT) and convert it to PWV to estimate blood pressure [12]. BPro uses tonometry to measure the time difference between the primary peak of the pulse wave and the second peak of the reflection [13]. Physiologically, the time difference between the second peak and the primary peak of pulse wave reflection will reflect the arterial stiffness, which can be used to estimate blood pressure [14]. Other studies also measure two points in a short distance of the artery and measure the local pulse transit time (PTT) difference to estimate blood pressure [15].

Although these methods can reduce the device’s size so that the sensor can integrate into the wearable device, they all use the measured values of BPM and PWV to build regression models or use some non-linear methods to map the PWV and blood pressure. The user must first input the blood pressure value of the BPM into the system and map the PWV and blood pressure by algorithms for calibration [16]. This calibration process is necessary for the current popular cuffless technology and needs to be performed periodically. In addition, the current cuffless wearable blood pressure measurement devices [17], [18], [19] use ECG and PPG sensors to measure PAT, which uses PAT and blood pressure for the regression model. However, studies have pointed out that PAT will be affected by the pre-ejection period (PEP) [20], [21] As a result, PAT cannot accurately estimate PWV and then estimate blood pressure. Although human blood pressure does not change drastically in a short time, using a PAT and BP regression model will cause many potential parameters to be ignored and lose accuracy. The MK equation [22] in hemodynamics describes the relationship between PWV and blood pressure. The characteristics of arterial parameters must be considered. Different individual arteries have different characteristics of hemodynamics. Therefore, only the mapping between PWV and blood pressure may be inaccurate. Many studies have used machine learning models in recent years, using various physiological signal characteristics and demographics (e.g., age, sex, weight) as parameters and matching different hyperparameters to predict BP. Although it is claimed that the cuffless system can obtain acceptable accuracy under calibration-free, it is unclear how much of the attained BP measurement accuracy is due to the actual hemodynamic measurement [23]. The current machine learning model has insufficient interpretability for BP estimation, so regulatory agencies in various countries cannot approve this type of cuffless BPM. Based on hemodynamic cuffless BPM, calibration-free can also accurately estimate BP. The feasible way in the current research is to measure the artery parameters of different individuals, combine the measured PWV, and modify the BP estimation method based on the MK equation. For example, use two PPG probes to detect individual PWV [24] and set up an ultrasonic probe between the two PPG probes to measure the participant’s arterial pressure waveforms, simultaneously acquired arterial dimensions, and local PWV. Perform calibration-free cuffless BP estimation based on the measured data [25]. This technology has been able to realize calibration-free cuffless BP estimation based on hemodynamics. However, it needs to be measured by professionals in operation, and it is not easy to apply to long-term BP monitoring scenarios. Therefore, we propose a low-cost and small-sized PWV measurement device, which is used with color ultrasound imaging equipment to obtain the arterial parameters of the participant and combines the two to estimate BP with a hemodynamic mathematical model. As a pilot study, we studied the participants who need to perform long-term BP monitoring records in the future. Before collecting long-term BP, use color ultrasound imaging equipment to determine the participant’s arterial parameters, then combine the small wearable device proposed in this study with the hemodynamic mathematical model to estimate BP. Finally, we discussed the accuracy and future works that this method needs to be improved.

## II. Our Contribution

The contribution of this research is to propose a small, low-cost local PWV sensor and design algorithm to analyze pulse waves to estimate BP. We use color ultrasound imaging equipment to measure the arterial parameters required by the MK equation. These arterial parameters, along with the PWV measured by the sensor, are used to estimate BP using the MK equation. Also, we follow the ISO 81060–2 standard and the Bland–Altman plot statistical method to analyze data from 32 participants. We compare some cuffless technical characteristics in Table 1, including the currently popular cuffless methods [26] and potentially feasible calibration-free cuffless methods [27]. The advantages of our method are that it does not use intermittent inflation BPM for calibration and has the potential for beat-to-beat BP monitoring. Finally, according to the results of our experiments, this pilot study verifies that the measurement method of the cuffless BPM we proposed can estimate BP calibration-free. It provides a more convenient method for future calibration-free cuffless BPM research work.

**TABLE 1:** Compare the Current Popular Cuffless Technology and Electronic BP Monitor

	Our proposed method	Oscillometric finger pressing method	Popular cuffless BP monitor	Electronic BP monitor
Sensing technology	Pulse wave sensor (Ultrasound image aid)	Pressure and PPG sensor	ECG and PPG sensor	Arterial pressure sensor
Measurement parameter	Pulse transit time	Pressure curve and optical envelope of heart beats	Pulse arrival time	The heart beats envelope curve
Principle	MK equation (with artery parameter)	Oscillometry method	Regression model	Oscillometry method
Calibration*a	No	No	Necessary	No
Beat-to-beat	Yes*b	No	Yes*b	No
Cuffless	Yes	Yes	Yes	No
Intermittent inflation	No	No	No	Necessary
Reliability	Good	Depends on the operation of the user	Influenced by pre-ejection period	Good
Measurement time	Beat-to-beat	Depends on the operation of the user	Beat-to-beat	30 to 40 seconds

^*a.^ According to the IEEE 1708 standard, an additional BP measuring device is required to reference the calibration point, usually an electronic BP monitor compliant with the ISO 81060–2 standard.

^*b.^ It is technically feasible, but it is still necessary to average several data to reduce errors.

## III. Local PWV Sensor and Analog Front End(AFE) Design

Piezoelectrics are able to convert mechanical waves into electrical signals. Piezoelectric ceramic is a highly efficient electromechanical transducer that can sense weak vibrations through the direct piezoelectric effect [28]. We developed a low-cost glued and airtight packaging method to use piezoelectric ceramics to sense arterial pulse waves. The piezoelectric is sealed in an airtight cavity, and the sensor surface is placed in contact with the radial artery. When the aortic valve opens, blood is ejected into the aorta, and the pulse wave generates tension around the artery wall [29]; the pressure generated by this tension is detected by the deformation of the compression sensor surface. The airtight cavity in the sensor pressurizes in response to this deformation, in turn deforming the piezoelectric sensor and generating an AC signal that can be measured. The PWV can then be determined by measuring the difference in pulse arrival time between two such sensors. We use a mechanical model to analyze the mechanical structure of the sensor proposed in this study.

### A. Sensor Design

**FIGURE 1.**
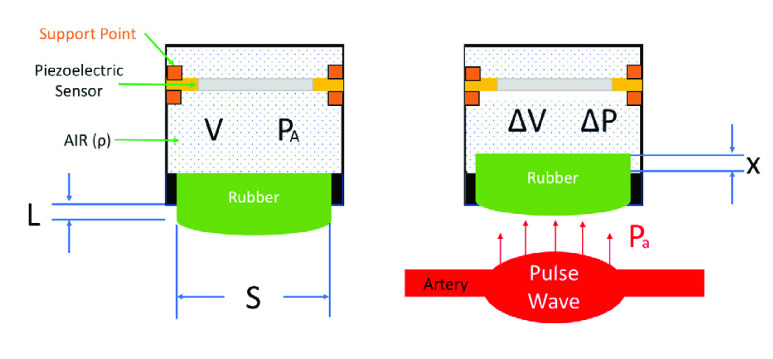
The sensor package.

A schematic of the sensor package is shown in Fig. 1. The piezoelectric ceramic is encapsulated in a cavity, and the surface in contact with the radial artery has a layer of the compressible rubber film. The volume of rubber film can be expressed according to Eq. 1, where $m$ is the mass of rubber, $\rho $ is the density of air, $L$ is the effective length and $S$ is the cross-sectional area. The volume of the cavity is $V$, and the atmospheric pressure is $P_{A}$. When the radial artery pulse wave reaches the contact surface of the sensor, the rubber film will be compressed by the distance $x$. As described in Eq. 2, the volume will decrease by the product of the cross-sectional area $S$ and the compression distance $x$. This compression will increase the pressure in the cavity by an amount of $P_{a}$ (Eq. 3).

**FIGURE 2.**
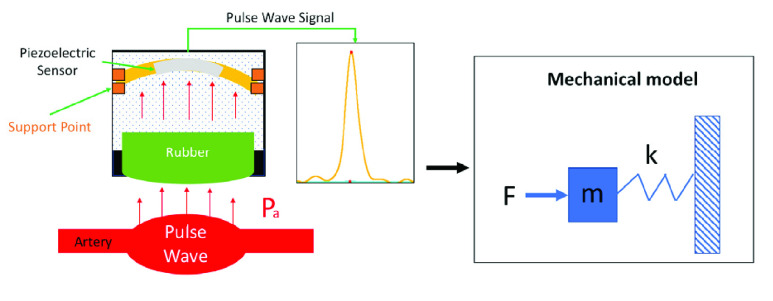
The pressure increase will determine the deformation of the piezoelectric.

When the pressure in the cavity increases, the pressure of the gas on the piezoelectric increases. The piezoelectric sensor packaged inside the cavity will then be deformed by the pressure generated during compression. The pressure increase will determine the deformation of the piezoelectric (Fig. 2), which is mathematically equivalent to the mass-on-a-spring system shown in Fig. 2. The force $F$ pushes the rubber film $m$, and the compressed gas $k$ in the cavity causes the piezoelectric to be deformed; the piezoelectric effect then generates an electrical signal. The electrical signal generated is equivalent to a direct conversion of the mechanical wave propagating along the artery [30].

The proportional relationship between the volume and pressure changes is described by Eq. 4, because the rubber film is squeezed by the pulse of the radial artery so that the internal pressure increases by $P_{a}$. The pressure change $P_{a}$ is produced by a small volume change $\Delta V$, and the resulting equation involves a constant, $\gamma $, the ratio of specific heats, which is approximately 1.4 for air [31]. Newton’s law of acceleration is Eq. 5. We can find the relationship between the force $F$ and the action force of the piezoelectric ceramic with Eq. 6.

\begin{align*} m&=\rho SL \tag{1}\\ \Delta V&=V-Sx \tag{2}\\ \Delta P&=P_{A} +P_{a} \tag{3}\\ \frac {P_{a}}{P_{A}}&=-\gamma \frac {\Delta V}{V}=-\gamma \frac {Sx}{V} \tag{4}\\ \frac {F}{m}&=\frac {d^{2}x}{dt^{2}} \tag{5}\\ \frac {d^{2}x}{dt^{2}}&=\frac {P_{a} S}{SL\rho }=-\frac {\gamma SP_{A}}{\rho VL}x \tag{6}\end{align*}

### B. Analog Front End Design

**FIGURE 3.**
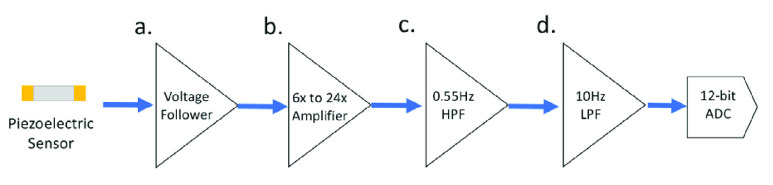
The piezoelectric sensor. a) The piezoelectric is connected to the input of the voltage follower. b) The output of the VF connects to the amplifier circuit. c) The output signal of the amplifier is passed through a high-pass filter. d) High-frequency AC signals above 10 Hz are attenuated by a low-pass filter.

The flowchart in Fig. 3 shows the relationship between the piezoelectric sensor and AFE. The signal output of the AFE is sampled by the 12-bit analog-to-digital converter (ADC). The ADC is integrated in the STM32F4 microcontroller, and the microprocessor runs the digital signal processing algorithm in real-time. Samples the pulse wave signal at a rate of 10 kHz, which can achieve a PTT measurement accuracy of 0.1 milliseconds.

**FIGURE 4.**
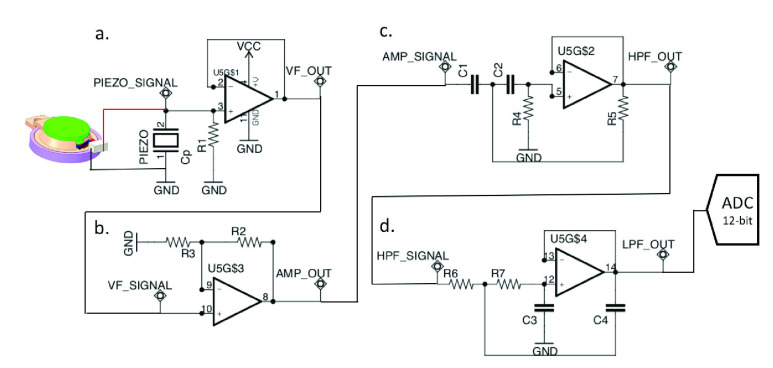
(a) VF and (b) signal amplifier and gain adjust. (c) Second-order Sallen–Key HPF and (d) second-order Sallen–Key LPF.

Fig.4a shows the voltage follower (VF), providing the complete input of the piezoelectric sensor signal. If we connect the piezoelectric sensor to the external load, we get a high-pass filter (HPF). The cut-off frequency of the HPF is the point in the circuit with a 3dB drop in magnitude. Therefore, we need to add an external load resistor to ensure the quality of the signal. To determine the corresponding load resistor R1 in Eq.7, we need to measure the parasitic capacitance Cp of the piezoelectric sensor [32], and f is the cut-off frequency of 1 Hz of heartbeats with rates around 60 beats per second of an average. The Cp of the piezoelectric ceramic we chose is 8000 pF ±30%. Therefore, the load resistance range can be calculated to be about 15M ohms to 28M ohms. Fig.4b is the signal amplifier. Eq.8 selects R2 and R3 to adjust the gain. The signal passes through the signal amplifier, and the pulse wave signal is amplified to hundreds of millivolts. When the signal of the pulse wave is amplified, the noise will also be amplified. Fig.4c is the second-order Sallen-Key HPF; Eq.9 is its transformation function, and the cut-off frequency is determined by Eq.10. This study’s HPF cut-off frequency set is 0.55 Hz; R4 and R5 are equal, and C1 and C2 are also designed to be the same value. After the signal passes through the HPF, the DC component can be filtered out. Fig.4d is the second-order Sallen-Key low-pass filter (LPF) [33], [34]; Eq.11 is its transform function, and the cut-off frequency is determined by Eq.12. In this study, the cut-off frequency of LPF is 10 Hz; R6 and R7 are equal, and C3 and C4 are also equal. They can attenuate the interference caused by the supply mains frequency and other disturbances.

\begin{align*} R1&=\frac {1}{(2\cdot \pi \cdot f\cdot C_{p})} \tag{7}\\ Vout&=Vin\cdot \frac {R2}{R3} \tag{8}\\ \frac {Vout(s)}{Vin(s)}&=\frac {s^{2}}{s^{2}+s\left ({{\frac {1}{R4\cdot C1}+\frac {1}{R4\cdot C2}} }\right)+\frac {1}{R4\cdot C1\cdot R5\cdot C2}} \tag{9}\\ fc_{HPF} &=\frac {1}{2\pi \sqrt {R4\cdot C1\cdot R5\cdot C2}} \tag{10}\\ \frac {Vout(s)}{Vin(s)}&=\frac {\frac {1}{R6\cdot C3\cdot R7\cdot C4}}{s^{2}+s\left ({{\frac {1}{R7\cdot C3}+\frac {1}{R6\cdot C3}} }\right)+\frac {1}{R6\cdot C3\cdot R7\cdot C4}} \tag{11}\\ fc_{LPF} &=\frac {1}{2\pi \sqrt {R6\cdot C3\cdot R7\cdot C4}} \tag{12}\end{align*}

### C. Local PWV Measurement

**FIGURE 5.**
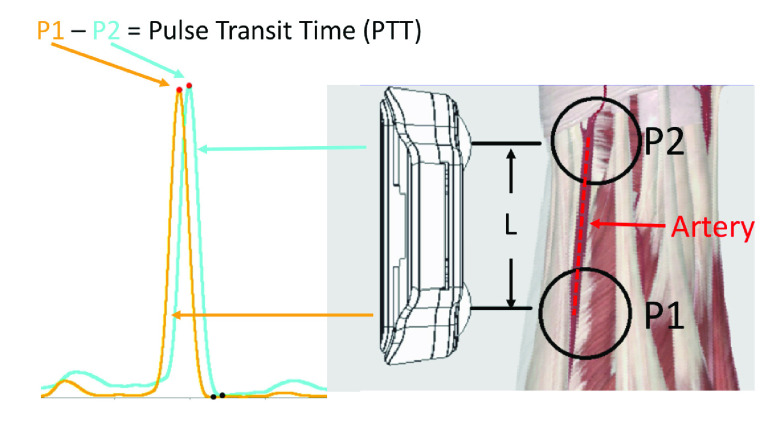
In this study, the local PWV was measured on the radial artery.

The local PWV is the pulse wave time difference generated by two sensors on the same arterial segment, separated by a small distance. In this study, the local PWV was measured on the radial artery. Two sensors were connected to two AFEs and placed on the radial artery at a fixed separation distance. A schematic of the device is shown in Fig. 5. When the pulse wave is transmitted from **P1** to **P2**, the time difference between the peaks of the AFE output at **P1** and **P2** can be measured to obtain the pulse transit time (PTT) [35]. The distance $L$ between **P1** and **P2** is fixed, and $L$ divided by the PTT gives the PWV. The shorter the PTT, the higher the blood pressure.

### D. Pulse Wave Signal Processing

The AFE output signal was sampled using a 12-bit ADC and then pre-processed using a finite impulse response (FIR) digital filter [36]. As shown in Eq.13, the pulse wave signal x sampled by the ADC is convolved with the filter coefficient bk to obtain the filtered pulse wave signal y [37]. As in Eq.14, the filter coefficients need to calculate the frequency response and window function coefficients according to different FIR filter types and the size of the convolution kernel. The related research on pulse wave analysis shows that the pulse wave frequency ranges from 0.8Hz to 30Hz [38], [39], [40], [41]. Therefore, we use a bandpass filter (Eq.15) and set the cutoff frequencies fc1 and fc2 to 0.6 Hz and 5 Hz, respectively. Since the pulse wave represents the heartbeat signal generated by the human body, periodic or quasi-periodic, the range of 0.6Hz to 5Hz represents 36 to 300 beats per minute, this frequency range includes the normal heartbeat range [42], [43], so this study selected this range as the cutoff frequency. Where $\omega $c1 and $\omega $c2 are the cutoff frequencies of the bandpass filter, and fc1 and fc2 are the transition frequencies, which need to be divided by the ADC sampling rate of 10kHz (Eq.16). The term w is the window function of the Kaiser window [44], as defined in Eq.17, and N is the window size chosen to be 128 in this study. The variable I0 is the second kind of modified Bessel function, as defined in Eq.18, where $\beta $ can adjust the frequency domain’s main lobe and sidelobe levels.

\begin{align*} y[n]&=\sum \limits _{k=0}^{N-1} {b_{k} \cdot x[n-k]} \tag{13}\\ b[n]&=w[n]\cdot h_{d} [n],0\le n\le N-1 \tag{14}\\ h_{d} [n]&=\left \{{{_{\frac {\omega _{c2} -\omega _{c1}}{\pi },n=\frac {N}{2}}^{\frac {\sin (\omega _{c2} (n-N))}{\pi (n-N)}-\frac {\sin (\omega _{c1} (n-N))}{\pi (n-N)},n\ne N}} }\right. \tag{15}\\ \omega _{c1} &=f_{c1} \frac {1}{f_{s}},\omega _{c2} =f_{c2} \frac {1}{f_{s} },f_{s} =10,000Hz \tag{16}\\ w[n]&=w_{0} \left ({{\frac {L}{N}\left({n-\frac {N}{2}}\right)} }\right) \\ &=\frac {I_{0} \left[{\beta \sqrt {1-\left({\frac {2n}{N}-1}\right)^{2}} }\right]}{I_{0} [\beta]},0\le n\le N \tag{17}\\ I_{0} &=\sum \limits _{i=1}^{n} {\left ({{\frac {(\beta \mathord {\left /{ {\vphantom {\beta 2}} }\right. } 2)^{i}}{i!}} }\right)^{2}} \tag{18}\end{align*}

Different participants may have different pulse wave amplitudes. Therefore, it is necessary to design a method that can dynamically adjust the threshold of the pulse wave detection algorithm. Eq.19 is the calculation method of dynamic threshold, and Eq.20 is the formula of covariance (CV) used to calculate the rate of change of the signal strength in the past. Before calculating CV, determine the mean value of the signal amplitude according to Eq.21. The root mean square (RMS) is the signal amplitude level according to Eq.22. It should be noted that the window size M for the dynamic threshold calculation is different from the FIR filter’s window size. This window’s size will affect the dynamic threshold’s response speed. In this research, the window size of the dynamic threshold calculation is set to be the same as the sampling rate, which can have an excellent dynamic adaptation to detect regular or irregular pulse waves. In our previous study [45], the window size could be set to half the sampling rate for patients with arrhythmia. The gain can adjust the dynamic threshold level, and we set it 1.1 times in this study. When the signal-to-noise ratio (SNR) is low, the waveform of the pulse wave has a smaller CV for the entire signal. Increasing the gain can reduce the false positive of peak detection.

\begin{align*} g\textrm {radient} &= \text {RMS}\cdot \frac {CV}{100}\cdot gain \tag{19}\\ CV&=\frac {\left ({{\sum \limits _{k=0}^{N-1} {x_{k} -mean}} }\right)^{2}}{M} \tag{20}\\ mean&=\frac {\sum \limits _{k=0}^{N-1} {x_{k}}}{M} \tag{21}\\ RMS&=\sqrt {\frac {\sum \limits _{k=0}^{N-1} {x_{k}^{2}}}{M}} \tag{22}\end{align*}

Algorithm (1) is the pseudocode [46] of the feature detection algorithm based on a dynamic gradient. When the signal amplitude starts to rise, the pulse wave is transmitted to the sensor. When the current signal amplitude is greater than the previous value, the local maximum is updated; the signal amplitude is less than the local maximum minus the gradient, and the previous local maximum is the peak of the pulse wave. The local minimum is updated when the signal amplitude decreases and the current amplitude is smaller than the previous value. The local minimum is the pulse wave trough when the signal amplitude is greater than the local minimum plus the gradient. The algorithm only needs to update the gradient and record the local maximum, local minimum, current state, and the previous state of Eq.19 to Eq.22, which can be used for real-time pulse wave detection. Moreover, this algorithm does not need to record part of the signal segment or signal data of a specific window size in the internal memory space. The current popular peak detection algorithm needs to store the signal. Need to calculate the mean and standard deviation and store the signal that the candidate features to further search for local peaks [47], [48], and an automatic multiscale-based peak detection (AMPD) algorithm [49], which requires a scaling window to detect signals stored in memory. The memory used by these algorithms varies with the signal’s sampling rate. In our proposed method, which only needs to store the state of the dynamic gradient calculation, the memory used is a fixed size, which is more suitable for running on wearable devices with limited memory and performance.

Algorithm 1 **Local Maximum and Minimum Detection Based on Dynamic Gradient**
Inputs: sensor signal{index, value}, and gradient
1: is_peak, is_trough: = FALSE
2: **if** value > local_maxima **then**
3: local_maxima: = signal{index, value}
4: **end if**
5: **if** value < local_minimal **then**
6: local_ minimal: = signal{index, value}
7: **end if**
8: **if** is_emission == **TRUE** and x < local_maxima
9: gradient **then**
10: is_emission: = **FALSE,**is_peak: = **TRUE**
11: falling: = signal{index, value}
12: local_minimal: = local_maxima
13: result: = {is_peak, local_maxima}
14: **end if**
15: **if** is_emission == **FALSE** and x > local_minimal +
16: gradient **then**
17: is_emission: = **TRUE,**is_trough: = **TRUE**
18: rising: = signal{index, value}
19: local_maxima: = local_minimal
20: result: = {is_trough, local_minimal}
**end if**
**if** is_ peak != **TRUE** and is_ trough != **TRUE then**
21: result: = {−1, the current signal is not a peak or trough }
22: **end if**
Outputs: result

**FIGURE 6.**
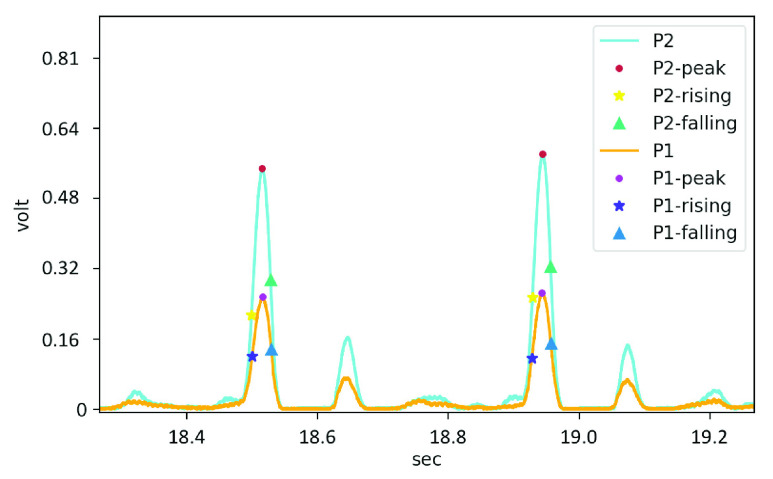
The digital filter and feature detection algorithm proposed in this research is applied to the measured pulse wave results.

**FIGURE 7.**
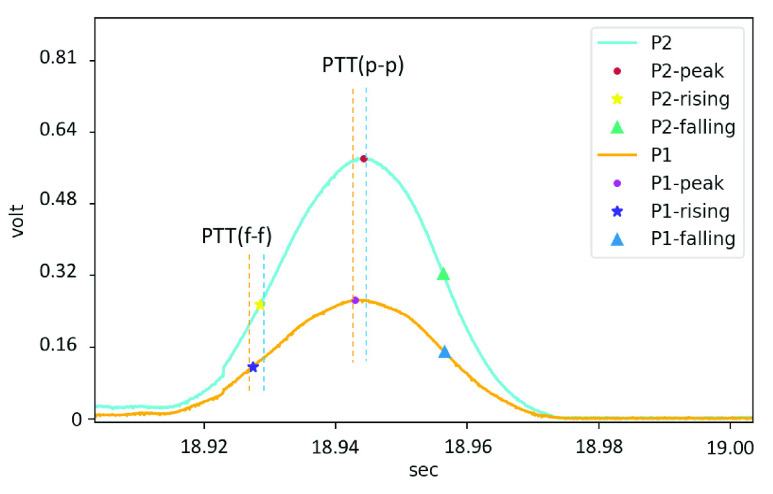
Peak-to-peak and foot-to-foot PTT detection.

The filtered pulse wave signal can be obtained, and apply the feature detection algorithm based on the dynamic gradient proposed in this research to label features, as shown in Fig. 6. The PTT was calculated from the relative time difference between the peak-to-peak (p-p) or foot-to-foot (f-f) [50] of the two pulse wave signals from **P1** and **P2**, as shown in Fig. 7.

## IV. Cuffless Blood Pressure Measurement

### A. Use PWV Blood Pressure Algorithm

The Bramwell-Hill (Eq. 23) and MK (Eq. 24,25) equations [51], based on hemodynamics, describe the basic principles widely used in cuffless BPM technology. The MK equation describes the relationship between PWV and BP, where $D$ is the diameter of the radial artery, $h$ is the wall thickness of the radial artery, $\rho $ is the average density of human blood (1.06 g/mL [52]), $E$ is Young’s modulus, $P$ is the pressure, and $E_{0}$ and $\gamma $ are coefficients with values of 1428.7 and 0.031 respectively [53]. $E_{0}$ and $\gamma $ depend on the stiffness of the radial artery and may be different in different individuals [54]. This study does not account for these differences, and uses constant $E_{0}$, $\gamma $, and $\rho $ values from previous studies to verify the BP measurement using local PWV. Ultrasound imaging was used to measure the radial artery diameter and artery wall thickness [55] of each participant, in order to verify the estimation of BP using the MK equation. PWV can be calculated from the measured PTT and the sensor separation distance, $L$, as described above (Eq. 26), and, according to Eq. 27, there is a non-linear function conversion between PWV and P. The pressure in this study is the radial artery pressure. Arterial pressure changes throughout the cardiac cycle. For the relationship between PWV and $P$, previous studies took $P$ as the mean arterial pressure (MAP). Since the systolic and diastolic phases take up approximately one third and two thirds of the cardiac cycle respectively [56], MAP is approximately equal to 1/3 multiplied by the systolic BP plus 2/3 multiplied by the diastolic BP (Eq. 28).

\begin{align*} PWV&=\sqrt {\frac {V\cdot dP}{\rho \cdot dV}} \tag{23}\\ PWV&=\sqrt {\frac {E_{inc} \cdot h}{D\cdot \rho }} \tag{24}\\ E_{inc} &=E_{0} \cdot \exp ^{\xi \cdot P} \tag{25}\\ PWV&=\frac {L}{PTT} \tag{26}\\ P&=\frac {\ln \left({\frac {PWV^{2}\cdot \rho \cdot D}{h\cdot E_{0}}}\right)}{\xi } \tag{27}\\ MAP&\approx \frac {SBP}{3}+\frac {DBP\cdot 2}{3} \tag{28}\end{align*}

### B. Artery Parameter and Blood Pressure Measurement

In previous studies, the arterial diameter and arterial wall thickness of the Moens-Korteweg equation were assumed to be constant [57], and regression models were used to simplify the equation to estimate the MAP, SBP, and DBP [58]. The problem with this method is that individual differences in arterial parameters may reduce the accuracy of the regression model, such that the blood pressure measurements fail to meet the ISO 81060–2 criteria of a mean difference of less than 5 mmHg and a standard deviation of less than 8 mmHg when compared to an aneroid sphygmomanometer.

In this study, the participant’s wrist was fixed to a holder, and the researcher operated the ultrasound imaging equipment to measure the radial artery. We used the measured diameter and wall thickness of each participant as the $D$ and $h$ parameters of the MK equation, and used the local PWV sensor proposed above to calculate the MAP.

## V. Experiment Results and Analysis

**FIGURE 8.**
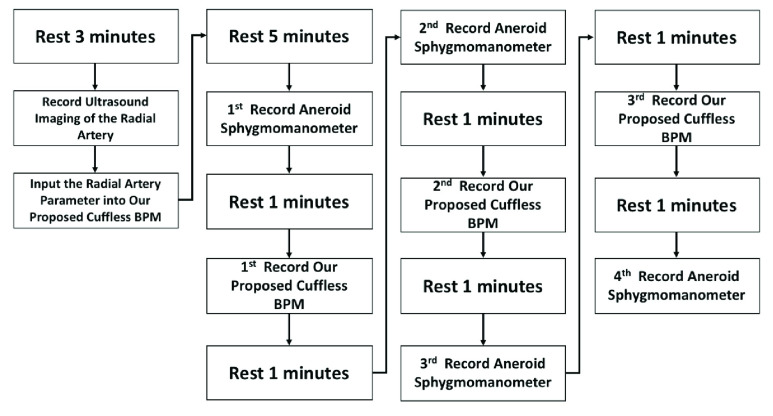
The flowchart of acquiring color ultrasound images of the participant’s radial artery and ISO 81060–2 protocol for non-invasive sphygmomanometers.

Before executing the ISO 81060–2 protocol, this study used ultrasound imaging equipment to measure the participant’s radial artery parameters. Then use our calibration-free cuffless BPM (Accurate24), combined with radial artery parameters and a mathematical model to estimate BP. As shown in the flowchart in Fig. 8, participants need to perform reference BPM (aneroid sphygmomanometer) four times to measure BP and average two adjacent reference BP to get a total of three averaged reference BP. Accurate24 performs three BP measurements. Finally, we will perform a Bland-Altman analysis to evaluate the performance of the calibration-free cuffless BPM.

### A. Ultrasound Imaging of the Radial Artery

**FIGURE 9.**
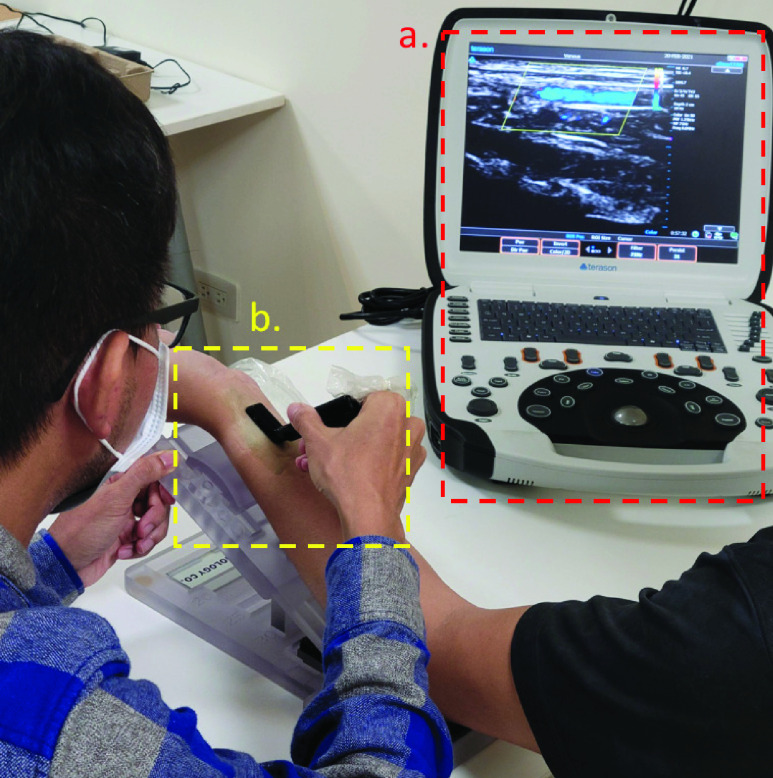
a) The portable ultrasound scanner uSmart-3300 produced by Terason. b) The L-Type ultrasound probe used to collect the ultrasound image of the participant’s radial artery.

Radial artery parameters can be measured using ultrasound imaging equipment [59]. We used the uSmart-3300 portable ultrasound scanner (Fig. 9a) and the L-type probe (Terason-16HL7), both produced by Terason, to measure the radial artery parameters of each participant. The participant’s left arm was placed on a 30° inclined platform, the position of the wrist was fixed, and the researcher operated the ultrasound probe to collect an image of the participant’s radial artery (Fig. 9b). The researcher who collected these data has good clinical practice (GCP) certification for the portable ultrasound scanner, and is qualified to make ultrasound readings for clinical trials.

The MK equation of the hemodynamics model assumes an ideal circular and straight cylinder. So we choose different measurement points on the radial artery segment. The average diameter and wall thickness measured at each point is the artery parameters for the entire artery segment.

**FIGURE 10.**
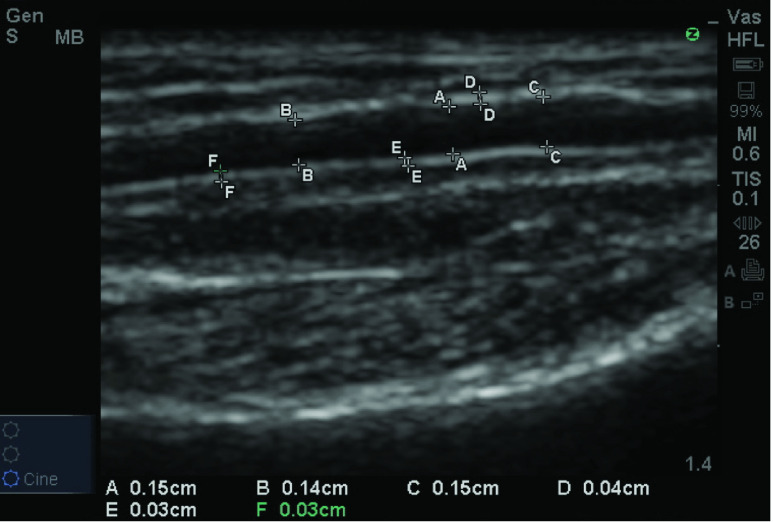
Labels A, B, C are artery diameters; Labels D, E, F are intima-media thickness.

As shown in Fig. 10, we used the L-Type ultrasound probe to measure the participant’s radial artery [60]. We placed the ultrasound probe against the contact surface without pressure between 2 cm above the styloid process and 6 cm above the styloid process [61]. We reference the method of assessing radial artery in systemic disease to measure the arterial diameter and intimal thickness [62].

### B. Local Pulse Wave Velocity Measurement

**FIGURE 11.**
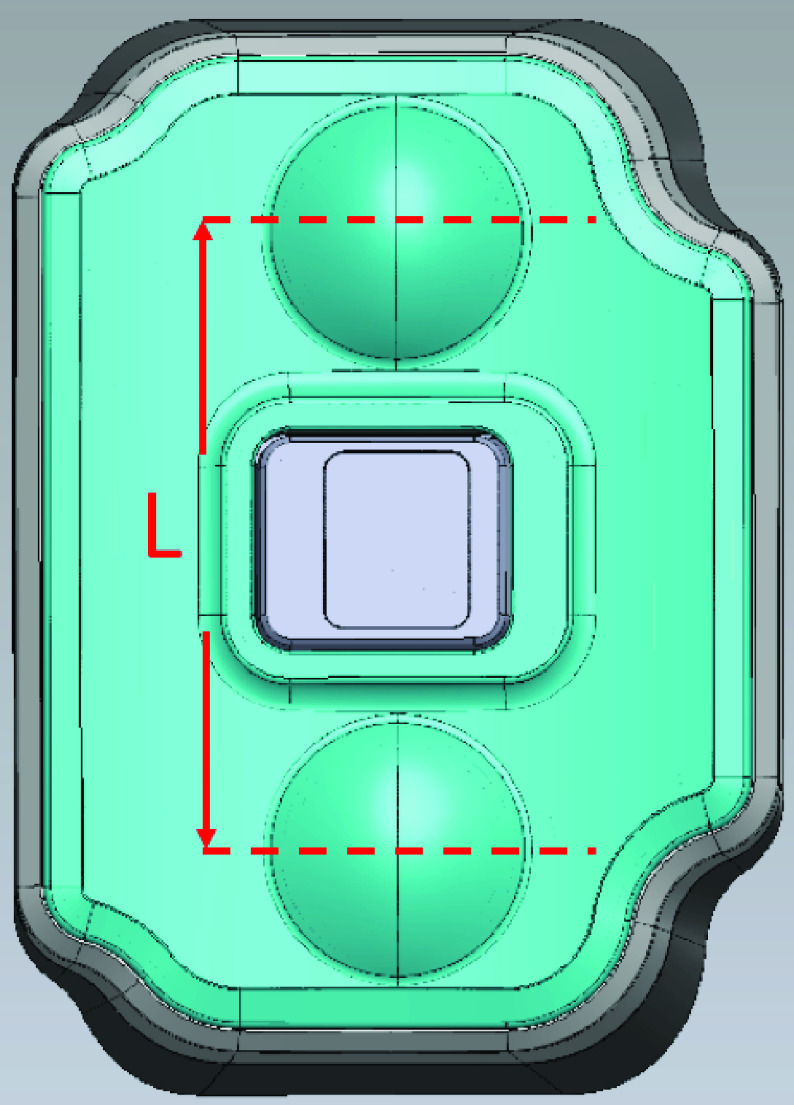
The measurement of local PWV uses the PTT between two measurement points on the artery.

**FIGURE 12.**
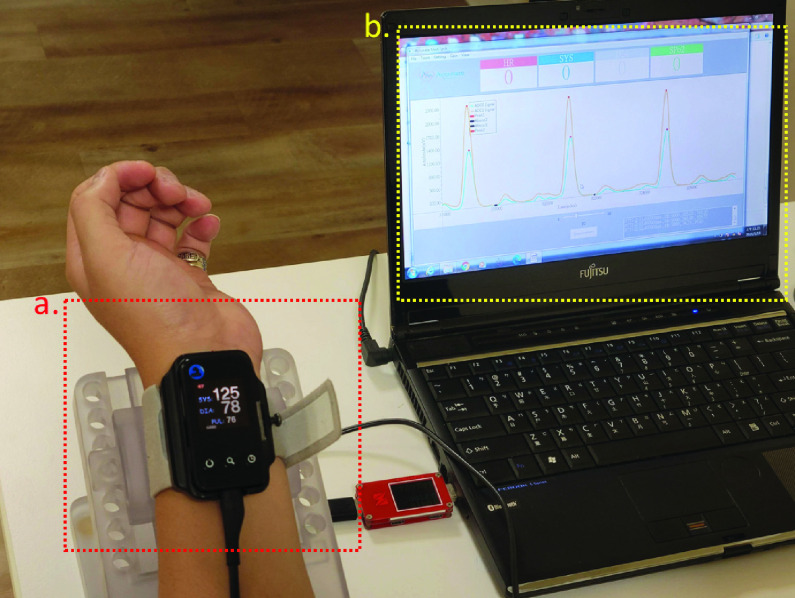
a) A device developed as part of this study, capable of measuring the user’s PWV in each cardiac cycle. b) Each pulse wave signal between the two sensors generates a local PWV.

The measurement of local PWV uses the PTT [63] between two measurement points on an artery. Since the distance between the two measurement points in our sensor was fixed at 3 cm (Fig. 11), the PTT could be used to directly calculate the local PWV. We used Accurate24 (Fig. 12a), a novel device developed as part of this study, to measure the participants’ PWV in each cardiac cycle, as described above. The two sensors of the Accurate24 measure the local PWV of the participant, as shown in Fig. 12b. It only needs to be fixed on the contact surface, and the pulse wave signal can be sensed without pressure. The dynamic threshold algorithm can track the pulse wave and detect the feature points after the participant stands still for one second. For each cuffless measurement in this study, we collected the 30-second cardiac cycle of the participants. A participant with a heartbeat of 60 beats per second can obtain 30 PWV data sets. We calculate the median of the PWV data set as the participant’s PWV. The participant placed their left arm on a 30° inclined platform, and the position of the wrist was fixed at the level of the heart. The researcher used the PC-host software to connect to the Accurate24 and collect the participant’s radial artery PTT, divide PTT by the distance $L$ between the two sensors, convert it to the local PWV, and record it. Accurate24 will estimate the BP value based on the participant’s arterial parameters and PWV.

### C. Blood Pressure Measurement

**FIGURE 13.**
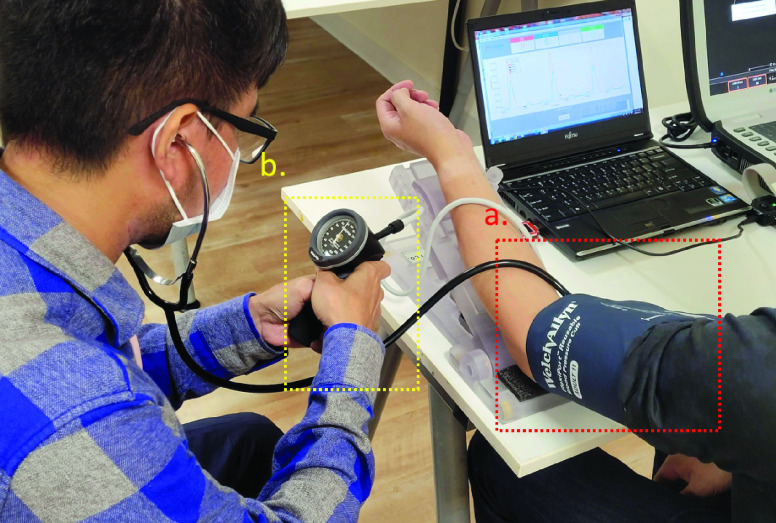
Welch Allyn 5098–27 DS66 trigger aneroid sphygmomanometer. a) The participant’s left arm is cuffed, and a stethoscope is placed inside the cuff. b) A pressure gauge is used to read the participant’s systolic BP.

The Korotkoff sounds method is the gold standard [64] for non-invasive BP measurement. The participant’s left arm is cuffed, and a stethoscope is placed inside the cuff (Fig. 13a). The cuff is pressurized until the arterial pulse cannot be heard. The vent valve releases gas at a rate of 3 to 5 mmHg per second. When the first sound of the pulse beating is heard, the reading on the pressure gauge (Fig. 13b) is the participant’s systolic BP. When the sound of pulse beating is no longer audible, the reading on the pressure gauge is the diastolic BP of the participant. The experiment setup is shown in Fig. 13. We used a Welch Allyn 5098–27 DS66 trigger aneroid sphygmomanometer.

We sampled the participant’s arterial parameters, used Accurate24 to sample the participant’s local PWV, used the MK equation to calculate BP, and compared the BP calculated from the MK equation to that measured using the aneroid sphygmomanometer. Since the aneroid sphygmomanometer can only measure SBP and DBP, we used Eq. 29 to calculate MAP [65]. Conversely, the pressure calculated by the MK equation approximates MAP, so we used Eq. 30 to estimate the SBP and DBP from the calculated MAP [66]. In this study, we used a $k$ factor of 0.76. We then compared the BP measured by the aneroid sphygmomanometer with the BP calculated using the MK equation.

\begin{align*} &MAP =\frac {SBP+(2\cdot DBP)}{3} \tag{29}\\ &\begin{cases} {DBP=MAP\cdot k} \\ {SBP=MAP+(1-k)\cdot DBP} \\ \end{cases} \tag{30}\end{align*}

### D. Result

**FIGURE 14.**
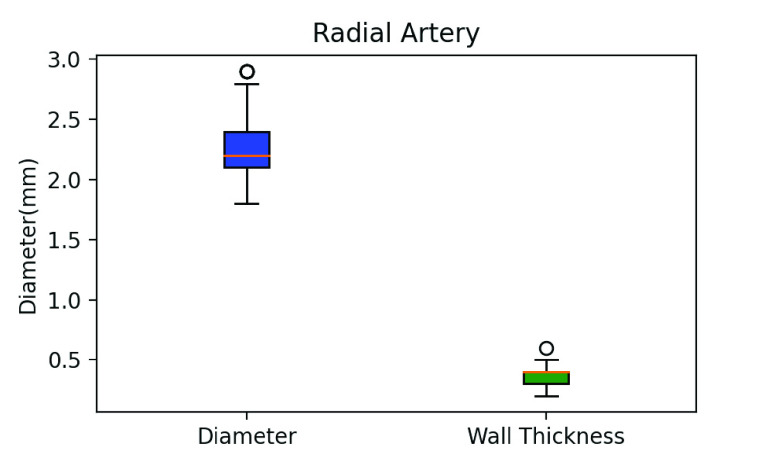
Box plot of the radial artery diameter and wall thickness.

**FIGURE 15.**
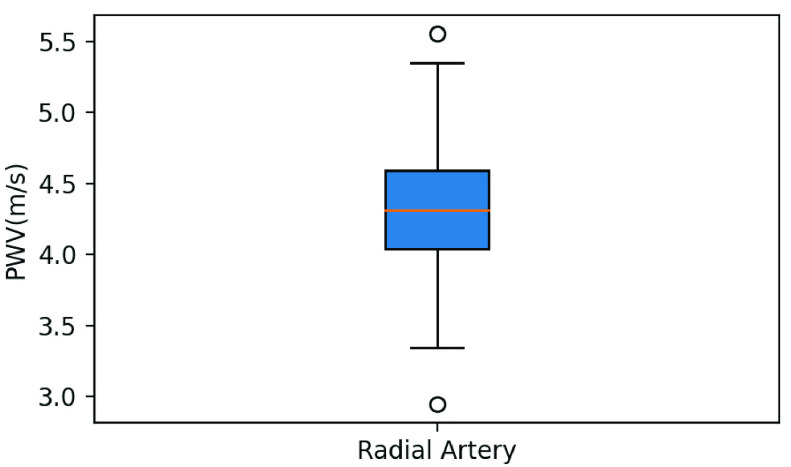
Box plot of the PWV of the participants measured by the sensor.

**FIGURE 16.**
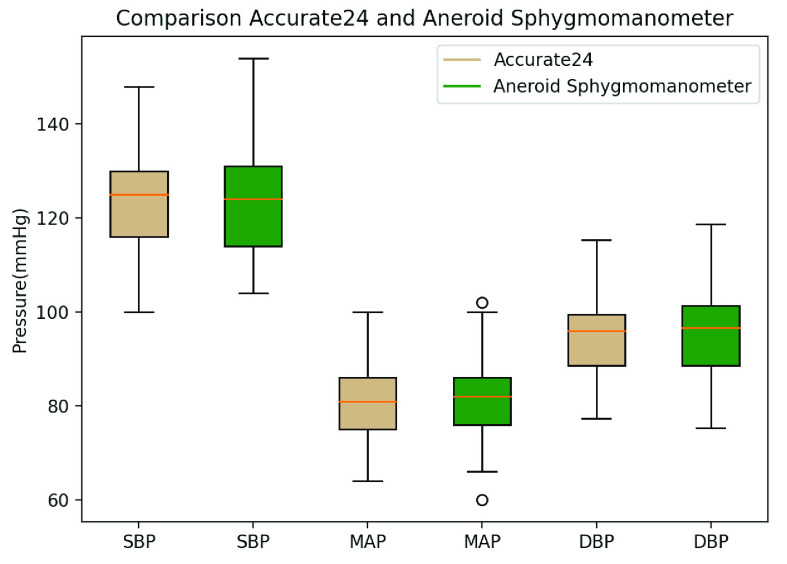
Box plot of BP of 32 participants, using MK equation to estimate BP, compared with reference BP. SBP– systolic blood pressure, MAP– mean arterial pressure, DBP– diastolic blood pressure.

Table 2 is the statistical data of the physiological characteristics of the 32 participants, including basic information, arterial parameters, and BP measured by both local PWV (Accurate24), and an aneroid sphygmomanometer. Fig. 14 and Fig. 15 are boxplots of the radial artery diameter, the wall thickness, and the PWV of the participants, respectively. Fig. 16 shows a box plot of BP calculated using the MK equation, and the BP measured by an aneroid sphygmomanometer.

**TABLE 2:** Subject Characteristics

	Subjects (n = 32)
Gender (M/F, n)	19/13
Mean age (range)	43 (23-74)
Artery diameter (mm)	2.35±0.55
Artery wall thickness (mm)	0.4±0.2
PWV (m/s)	4.25±1.33
ReferenceSBP (mmHg)	129±25
ReferenceMAP (mmHg)	97±22
ReferenceDBP (mmHg)	81±21
Accurate24SBP (mmHg)	124±24
Accurate24MAP (mmHg)	96±19
Accurate24DBP (mmHg)	82±18

ISO 81060–2 is a non-invasive electronic sphygmomanometer clinical standard. The standard requires that the mean difference between the BP measured by the tested sphygmomanometer and the reference BP measured by the aneroid sphygmomanometer should be less than 5 mmHg. In addition, the standard deviation should be less than 8 mmHg. The aneroid sphygmomanometer and the tested sphygmomanometer need to be used alternately 4 and 3 time respectively. The $i^{\mathrm {th}}$ measurement from the tested sphygmomanometer needs to be compared with the average value of the $i^{\mathrm {th}}$ and *i+1* $^{\mathrm {th}}$ measurements of the aneroid sphygmomanometer. The mean difference and standard deviation are calculated according to Eq. 31 and Eq. 32:

\begin{align*} md&=\frac {1}{n}\cdot \sum \limits _{i=1}^{n} {(BP_{SUT_{i}} -BP_{REF_{i}})} \tag{31}\\ std&=\sqrt {\frac {1}{n}\cdot \sum \limits _{i=1}^{n} {(BP_{SUT_{I}} -BP_{REF_{i} })^{2}}} \tag{32}\end{align*}

where $BP_{SUT_{I}} $ is the BP measured by the tested sphygmomanometer and $BP_{REF_{i}} $ is the reference BP measured by the aneroid sphygmomanometer.

**FIGURE 17.**
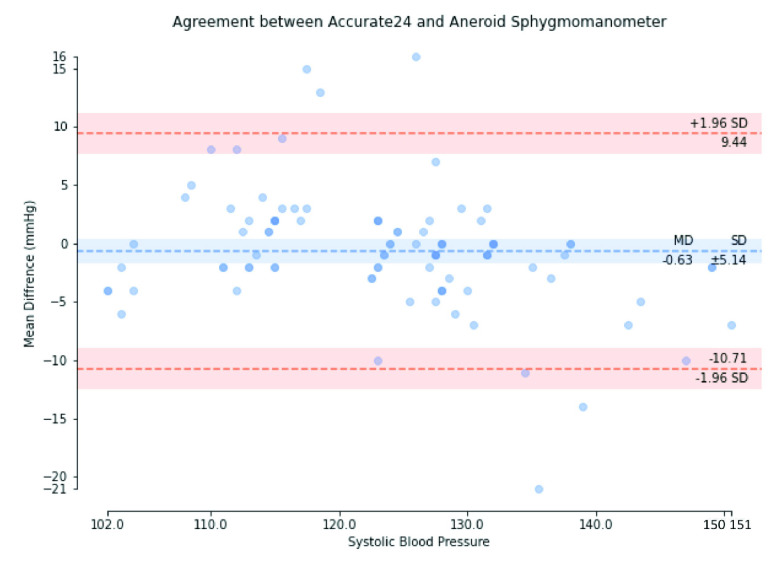
Bland-Altman plot of systolic blood pressure.

**FIGURE 18.**
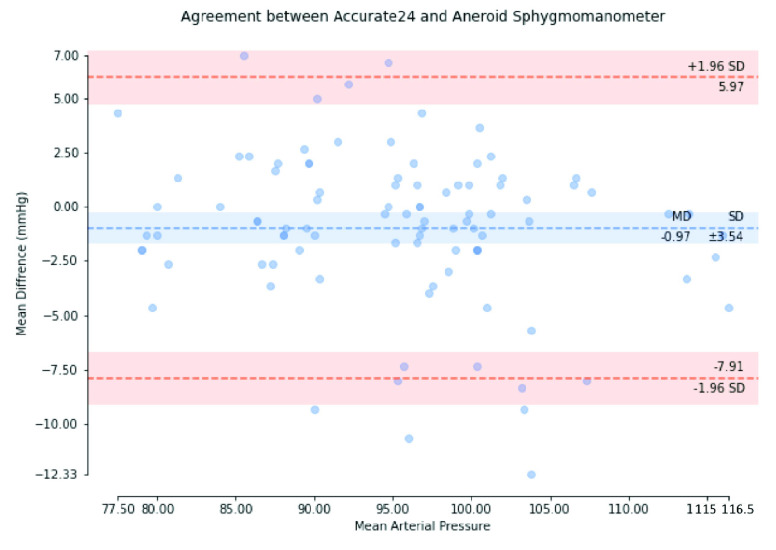
Bland-Altman plot of mean arterial pressure.

**FIGURE 19.**
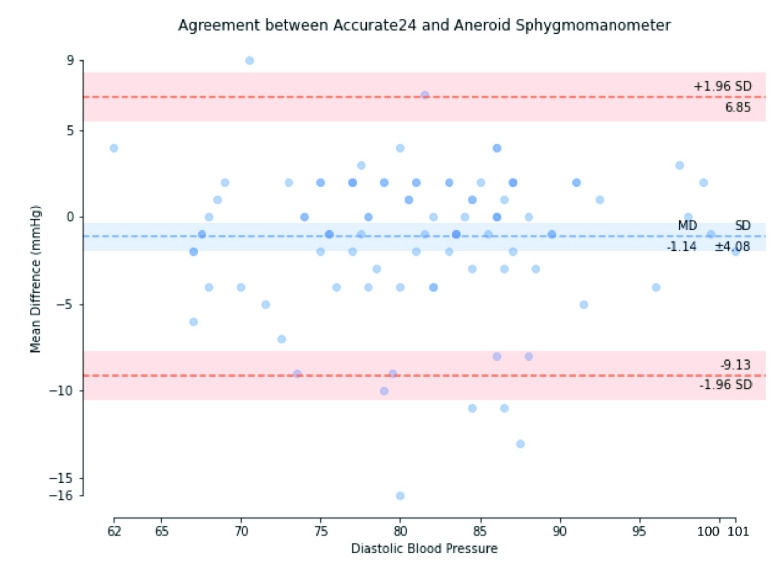
Bland-Altman plot of diastolic blood pressure.

We used the Bland-Altman plot statistical method [67] to compare the BP measured using Accurate24 and the aneroid sphygmomanometer. Fig. 17 shows the Bland-Altman plot of SBP, with a mean difference of −0.63 mmHg and a standard deviation of ±5.14 mmHg. Fig. 18 shows the Bland-Altman plot of MAP, with a mean difference of −0.97 mmHg, and a standard deviation of ±3.54 mmHg. Fig. 19 shows the Bland-Altman plot of DBP, with a mean difference of −1.14 mmHg, and a standard deviation of ±4.08 mmHg. These results demonstrate that the BP calculated using our method conforms to the ISO 81060–2 criteria of a mean difference ≤5 mmHg and a standard deviation ≤8 mmHg. Therefore, the calibration-free cuffless BPM proposed in this study complies with ISO 81060-2. This result indicates that this device could be used in a hospital intensive care unit (ICU) or in a remote health care setting, along with prior measurements of the patient’s arterial parameters, to perform beat-to-beat BP monitoring.

### E. Limitations and Future Works

Although the calibration-free cuffless BPM proposed in this study has verified its compliance with ISO 81060–2 through Bland-Altman analysis based on the experimental results of 32 participants, some limitations need further improvement for feasibility in long-term BP monitoring scenarios. First, for short-distance PWV measurement in the arterial segment, it is necessary to record a set of PWV and calculate the median as the PWV of the participant. It is because small changes in PTT will cause the error to be amplified when converting to PWV, which will cause a greater error when using hemodynamics to do BP estimation. The measurement techniques and algorithms must be optimized to reduce the error of beat-to-beat PTT measurement [68]. Secondly, the BP estimate of the MK equation is proximate to MAP. This study used the proportional coefficient to estimate SBP and DBP using MAP to get a larger standard deviation. It is because the pulse pressure (PP) of each individual is different. The PP of an individual with arteriosclerosis will be higher than that of a healthy person [69]. In addition to the BP estimation of the MK equation, it is still necessary to develop the technique of PP estimation. Combine the values of MAP and PP to make the estimation of SBP and DBP of calibration-free cuffless BPM more accurate. Finally, we still need to do more research on Young’s modulus of the MK equation. This coefficient makes the relationship between BP and PWV nonlinear. Although we use the coefficients obtained from previous animal experiments for calculations, different individuals have different arterial stiffness in practical applications. In the future, non-invasive equipment is needed to detect the participant’s arterial stiffness and improve the BP measurement accuracy of calibration-free cuffless BPM. Furthermore, after improving this research, the effective number of participants will be increased to 85 or more.

## VI. Conclusion

This study discusses the principles of current wearable cuffless BP measurement technology and describes the shortcomings that need to be addressed. We used a novel local PWV measurement sensor and ultrasound imaging equipment to collect participants’ arterial parameters and estimate their BP based on the Moens-Korteweg equation. In contrast to other cuffless BPM approaches, we explicitly account for individual differences in arterial parameters. We also verified that local PWV and ultrasound arterial parameter measurements can be used to directly calculate BP using the Moens-Korteweg equation. Furthermore, our proposed method does not require calibration against a standard BP measurement. Thus, this study demonstrates proof-of-principle for a novel non-invasive calibration-free cuffless BP estimate technology. Our piezoelectric sensor uses passive sensing without external power consumption. The size of the device can be small, which is suitable for long-term BP monitoring. The low-cost and highly sensitive sensor, which is conducive to integration into consumer electronic products such as wearable devices and remote health care monitors, could be used as a primary ambulatory BP monitoring tool in the future.

## Supplementary Materials

Supplementary materials

## References

[1] S. Shirmohammadi, K. Barbe, D. Grimaldi, S. Rapuano, and S. Grassini, “Instrumentation and measurement in medical, biomedical, and healthcare systems,” IEEE Instrum. Meas. Mag., vol. 19, no. 5, pp. 6–12, Oct. 2016.

[2] D. Dias and J. P. S. Cunha, “Wearable health devices—Vital sign monitoring, systems and technologies,” Sensors, vol. 18, no. 8, p. 2414, Jul. 2018. 30044415 10.3390/s18082414 PMC6111409

[3] M. S. Walid, S. N. Donahue, D. M. Darmohray, L. A. Hyer Jr., and J. S. Robinson Jr., , “The fifth vital sign—What does it mean?” Pain Pract., vol. 8, no. 6, pp. 417–422, Nov. 2008. 18662363 10.1111/j.1533-2500.2008.00222.x

[4] J. Jilek and T. Fukushima, “Oscillometric blood pressure measurement: The methodology, some observations, and suggestions,” Biomed. Instrum. Technol., vol. 39, no. 3, pp. 237–241, 2005. 15915608 10.2345/0899-8205(2005)39[237:OBPMTM]2.0.CO;2

[5] J. Sosa, “Wireless and batteryless blood pressure sensor,” in Proc. IEEE 54th Int. Midwest Symp. Circuits Syst. (MWSCAS), Aug. 2011, pp. 1–4.

[6] K. M. Borow and J. W. Newburger, “Noninvasive estimation of central aortic pressure using the oscillometric method for analyzing systemic artery pulsatile blood flow: Comparative study of indirect systolic, diastolic, and mean brachial artery pressure with simultaneous direct ascending aortic pressure measurements,” Amer. Heart J., vol. 103, no. 5, pp. 879–886, May 1982. 7072592 10.1016/0002-8703(82)90403-3

[7] G. Drzewiecki, R. Hood, and H. Apple, “Theory of the oscillometric maximum and the systolic and diastolic detection ratios,” Ann. Biomed. Eng., vol. 22, no. 1, pp. 88–96, Jan./Feb. 1994. 8060030 10.1007/BF02368225

[8] J. A. Staessen, “Task force II: Blood pressure measurement and cardiovacular outcome,” Blood Pressure Monitor., vol. 6, no. 6, pp. 355–370, Dec. 2001. 10.1097/00126097-200112000-00016 12055415

[9] S. Lee, H. R. Dajani, S. Rajan, G. Lee, and V.Z. Groza, “Uncertainty in blood pressure measurement estimated using ensemble-based recursive methodology,” Sensors, vol. 20, no. 7, p. 2108, Apr. 2020. 32276502 10.3390/s20072108 PMC7180780

[10] IEEE Standard for Wearable Cuffless Blood Pressure Measuring Devices, Standard 1708-2014, IEEE Standard Association, 2014.

[11] A. J. Viera, K. Lingley, and A. L. Hinderliter, “Tolerability of the Oscar 2 ambulatory blood pressure monitor among research participants: A cross-sectional repeated measures study,” BMC Med. Res. Methodology, vol. 11, no. 1, p. 59, Dec. 2011. 10.1186/1471-2288-11-59 PMC3097008 21524301

[12] N. Boubouchairopoulou, “A novel cuffless device for self-measurement of blood pressure: Concept, performance and clinical validation,” J. Human Hypertension, vol. 31, no. 7, pp. 479–482, Jul. 2017. 10.1038/jhh.2016.101 28124684

[13] S. Theilade, T. W. Hansen, C. Joergensen, M. Lajer, and P. Rossing, “Tonometric devices for central aortic systolic pressure measurements in patients with type 1 diabetes: Comparison of the BPro and SphygmoCor devices,” Blood Pressure Monitor., vol. 18, no. 3, pp. 156–160, 2013. 10.1097/MBP.0b013e328360fb19 23546451

[14] T. Tamura, “Cuffless blood pressure monitors: Principles, standards and approval for medical use,” IEICE Trans. Commun., vol. 104, no. 6, pp. 580–586, Jun. 2021.

[15] P. M. Nabeel, J. Joseph, and M. Sivaprakasam, “Magnetic plethysmograph transducers for local blood pulse wave velocity measurement,” in Proc. 36th Annu. Int. Conf. IEEE Eng. Med. Biol. Soc., Aug. 2014, pp. 1953–1956. 10.1109/EMBC.2014.6943995 25570363

[16] A. Steptoe, H. Smulyan, and B. Gribbin, “Pulse wave velocity and blood pressure change: Calibration and applications,” Psychophysiology, vol. 13, no. 5, pp. 488–493, 1976. 972973 10.1111/j.1469-8986.1976.tb00866.x

[17] I. Sharifi, S. Goudarzi, and M. B. Khodabakhshi, “A novel dynamical approach in continuous cuffless blood pressure estimation based on ECG and PPG signals,” Artif. Intell. Med., vol. 97, pp. 143–151, Jun. 2019. 30587391 10.1016/j.artmed.2018.12.005

[18] G. Zhang, S. A. McCombie, R. Greenstein, and D. B. McCombie, “Assessing the challenges of a pulse wave velocity based blood pressure measurement in surgical patients,” in Proc. 36th Annu. Int. Conf. IEEE Eng. Med. Biol. Soc., Aug. 2014, pp. 574–577. 10.1109/EMBC.2014.6943656 25570024

[19] A. Esmaili, M. Kachuee, and M. Shabany, “Nonlinear cuffless blood pressure estimation of healthy subjects using pulse transit time and arrival time,” IEEE Trans. Instrum. Meas., vol. 66, no. 12, pp. 3299–3308, Dec. 2017.

[20] J. Muehlsteff, X.L. Aubert, and M. Schuett, “Cuffless estimation of systolic blood pressure for short effort bicycle tests: The prominent role of the pre-ejection period,” in Proc. Int. Conf. IEEE Eng. Med. Biol. Soc., Aug. 2006, pp. 5088–5092. 10.1109/IEMBS.2006.260275 17946673

[21] S. L.-O. Martin, “Weighing scale-based pulse transit time is a superior marker of blood pressure than conventional pulse arrival time,” Sci. Rep., vol. 6, no. 1, pp. 1–8, Dec. 2016. 27976741 10.1038/srep39273 PMC5157040

[22] Y. Ma, J. Choi, A. Hourlier-Fargette, Y. Xue, H. U. Chung, and J. Y. Lee, “Relation between blood pressure and pulse wave velocity for human arteries,” Proc. Nat. Acad. Sci. USA, vol. 115, no. 44, pp. 11144–11149, 2018. 30322935 10.1073/pnas.1814392115 PMC6217416

[23] R. Mukkamala, “Evaluation of the accuracy of cuffless blood pressure measurement devices: Challenges and proposals,” Hypertension, vol. 78, no. 5, pp. 1161–1167, Nov. 2021. 34510915 10.1161/HYPERTENSIONAHA.121.17747 PMC8516718

[24] P. M. Nabeel, S. Karthik, J. Joseph, and M. Sivaprakasam, “Arterial blood pressure estimation from local pulse wave velocity using dual-element photoplethysmograph probe,” IEEE Trans. Instrum. Meas., vol. 67, no. 6, pp. 1399–1408, Jun. 2018.

[25] P. M. Nabeel, J. Jayaraj, K. Srinivasa, S. Mohanasankar, and M. Chenniappan, “Bi-modal arterial compliance probe for calibration-free cuffless blood pressure estimation,” IEEE Trans. Biomed. Eng., vol. 65, no. 11, pp. 2392–2404, Nov. 2018. 30130174 10.1109/TBME.2018.2866332

[26] R. Mukkamala, G. S. Stergiou, and A. P. Avolio, “Cuffless blood pressure measurement,” Annu. Rev. Biomed. Eng., vol. 24, no. 1, pp. 203–230, 2022. 35363536 10.1146/annurev-bioeng-110220-014644

[27] A. Chandrasekhar, C.-S. Kim, M. Naji, K. Natarajan, J.-O. Hahn, and R. Mukkamala, “Smartphone-based blood pressure monitoring via the oscillometric finger-pressing method,” Sci. Transl. Med., vol. 10, no. 431, p. 8674, Mar. 2018. 10.1126/scitranslmed.aap8674 PMC6039119 29515001

[28] D. Y. Park, “Self-powered real-time arterial pulse monitoring using ultrathin epidermal piezoelectric sensors,” Adv. Mater., vol. 29, no. 37, 2017, Art. no. 1702308. 10.1002/adma.201702308 28714239

[29] M. E. Safar, P. M. Nilsson, J. Blacher, and A. Mimran, “Pulse pressure, arterial stiffness, and end-organ damage,” Current Hypertension Rep., vol. 14, no. 4, pp. 339–344, Aug. 2012. 10.1007/s11906-012-0272-9 22555981

[30] J. Y. A. Foo and C. S. Lim, “Pulse transit time based on piezoelectric technique at the radial artery,” J. Clin. Monitor. Comput., vol. 20, no. 3, pp. 185–192, Jul. 2006. 10.1007/s10877-006-9019-y 16703422

[31] G. V. Shoev and M. S. Ivanov, “Numerical study of shock wave interaction in steady flows of a viscous heat-conducting gas with a low ratio of specific heats,” Thermophys. Aeromechanics, vol. 23, no. 3, pp. 343–354, May 2016.

[32] E. Bartolome and E. Bartolome, “Signal conditioning for piezoelectric sensors,” Texas Instrum. Dallas, TX, USA, Appl. Rep. SLOA033A, 2000.

[33] J. Karki, “Analysis of the sallen-key architecture,” Texas Instrum. Appl., Dallas, TX, USA, Tech. Rep. SLOA024B, 2002.

[34] J. Chen, P. Li, G. Song, Y. Tan, Y. Zheng, and Y. Han, “Systematic development of a wireless sensor network for piezo-based sensing,” J. Sensors, vol. 2018, pp. 1–12, Aug. 2018.

[35] P. M. Nabeel, J. Joseph, M. I. Shah, and M. Sivaprakasam, “Evaluation of local pulse wave velocity using an image free ultrasound technique,” in Proc. IEEE Int. Symp. Med. Meas. Appl. (MeMeA), Jun. 2018, pp. 1–6.

[36] Y. Lim and S. Parker, “FIR filter design over a discrete powers-of-two coefficient space,” IEEE Trans. Acoust., Speech, Signal Process., vol. ASSP-31, no. 3, pp. 583–591, Jun. 1983.

[37] G. Jinding, H. Yubao, and S. Long, “Design and FPGA implementation of linear FIR low-pass filter based on Kaiser window function,” in Proc. 4th Int. Conf. Intell. Comput. Technol. Autom., Mar. 2011, pp. 496–498.

[38] C. Chen, Z. Li, Y. Zhang, S. Zhang, J. Hou, and H. Zhang, “A 3D wrist pulse signal acquisition system for width information of pulse wave,” Sensors, vol. 20, no. 1, p. 11, Dec. 2019. 31861412 10.3390/s20010011 PMC6983233

[39] T. Panula, T. Koivisto, M. Pänkäälä, T. Niiranen, I. Kantola, and M. Kaisti, “An instrument for measuring blood pressure and assessing cardiovascular health from the fingertip,” Biosensors Bioelectron., vol. 167, Nov. 2020, Art. no. 112483. 10.1016/j.bios.2020.112483 32818750

[40] T. Nabeshima, T.-V. Nguyen, and H. Takahashi, “Frequency characteristics of pulse wave sensor using MEMS piezoresistive cantilever element,” Micromachines, vol. 13, no. 5, p. 645, Apr. 2022. 35630112 10.3390/mi13050645 PMC9144857

[41] J. McLaughlin, M. McNeill, B. Braun, and P. D. McCormack, “Piezoelectric sensor determination of arterial pulse wave velocity,” Physiol. Meas., vol. 24, no. 3, pp. 693–702, 2003. 14509307 10.1088/0967-3334/24/3/306

[42] A. D. Jose and D. Collison, “The normal range and determinants of the intrinsic heart rate in man,” Cardiovascular Res., vol. 4, no. 2, pp. 160–167, Apr. 1970. 10.1093/cvr/4.2.160 4192616

[43] T. Opthof and R. Coronel, “The normal range and determinants of the intrinsic heart rate in man,” Cardiovasc. Res., vol. 45, no. 1, pp. 175–176, 2000. 10728331 10.1016/s0008-6363(99)00321-1

[44] C. Yang and N. Tavassolian, “Pulse transit time measurement using seismocardiogram, photoplethysmogram, and acoustic recordings: Evaluation and comparison,” IEEE J. Biomed. Health Inform., vol. 22, no. 3, pp. 733–740, May 2017. 28436909 10.1109/JBHI.2017.2696703

[45] C.-Y. Guo, K.-J. Wang, and T.-L. Hsieh, “Piezoelectric sensor for the monitoring of arterial pulse wave: Detection of arrhythmia occurring in PAC/PVC patients,” Sensors, vol. 21, no. 20, p. 6915, Oct. 2021. 34696128 10.3390/s21206915 PMC8540434

[46] P. Spachos, J. Gao, and D. Hatzinakos, “Feasibility study of photoplethysmographic signals for biometric identification,” in Proc. 17th Int. Conf. Digit. Signal Process. (DSP), Jul. 2011, pp. 1–5.

[47] M. L. Jacobson, “Auto-threshold peak detection in physiological signals,” in Proc. 23rd Annu. Int. Conf. IEEE Eng. Med. Biol. Soc., vol. 3. Oct. 2001, pp. 2194–2195.

[48] G. K. Palshikar, “Simple algorithms for peak detection in time-series,” in Proc. 1st Int. Conf. Adv. Data Anal., 2009, pp. 1–13.

[49] F. Scholkmann, J. Boss, and M. Wolf, “An efficient algorithm for automatic peak detection in noisy periodic and quasi-periodic signals,” Algorithms, vol. 5, no. 4, pp. 588–603, 2012.

[50] P. C. Chang, A. Lin, G. A. Secor, and K. S. Su, “Determination of the pulse wave velocity by a filtered cross-correlation technique,” J. Biomechanics, vol. 4, no. 6, pp. 579–587, Dec. 1971. 10.1016/0021-9290(71)90047-9 5162579

[51] W. W. Nichols, McDonald’s Blood Flow in Arteries: Theoretical, Experimental and Clinical Principles, 6th ed. Abingdon, U.K: Taylor & Francis, 2011.

[52] M. Grumann, “Sensitivity enhancement for colorimetric glucose assays on whole blood by on-chip beam-guidance,” Biomed. Microdevices, vol. 8, no. 3, pp. 209–214, Sep. 2006. 16732473 10.1007/s10544-006-8172-x

[53] Y.-L. Zheng, B. P. Yan, Y.-T. Zhang, and C. C. Y. Poon, “An armband wearable device for overnight and cuff-less blood pressure measurement,” IEEE Trans. Biomed. Eng., vol. 61, no. 7, pp. 2179–2186, Jul. 2014. 24760899 10.1109/TBME.2014.2318779

[54] D. Hughes, C. F. Babbs, L. Geddes, and J. Bourland, “Measurements of Young’s modulus of elasticity of the canine aorta with ultrasound,” Ultrason. Imag., vol. 1, no. 4, pp. 356–367, 1979. 10.1177/016173467900100406 575833

[55] T. Dorman, “Ultrasound evidence of the optimal wrist position for radial artery cannulation,” Yearbook Crit. Care Med., vol. 2010, pp. 102–104, Jan. 2010.

[56] P. L.M. Kerkhof and V.M. Miller, Sex-Specific Analysis of Cardiovascular Function, 1st ed. Basel, Switzerland: Springer, 2018.

[57] H. Spahr, D. Hillmann, C. Pfäffle, and G. Hüttmann, “Comment on ‘Retinal pulse wave velocity measurement using spectral-domain optical coherence tomography,”’ J. Biophoton., vol. 11, no. 2, Feb. 2018, Art. no. e201700347. 10.1002/jbio.201700347 29219240

[58] W.-R. Yan, R.-C. Peng, Y.-T. Zhang, and D. Ho, “Cuffless continuous blood pressure estimation from pulse morphology of photoplethysmograms,” IEEE Access, vol. 7, pp. 141970–141977, 2019.

[59] V. Selvaraj and F. S. Buhari, “Ultrasound evaluation of effect of different degree of wrist extension on radial artery dimension at the wrist joint,” Ann. Card. Anaesth., vol. 19, no. 1, pp. 63–67, 2016. 26750676 10.4103/0971-9784.173022 PMC4900382

[60] J. C. Carretero, F. C. Montoya, M. G. Barez, M. Á.C. Montoya, M. O. Hernández, and R. Larrainzar-Garijo, “Description and analysis of the dynamic and morphological flow pattern of the main arteries of the wrist and hand in a healthy Spanish population,” Rev. Esp. Cir. Ortop. Traumatol., vol. 64, no. 3, pp. 167–176, 2020. 10.1016/j.recot.2019.12.004 32171673

[61] Z. Domagala, “Ultrasound evaluation of the radial artery in young adults—A pilot study,” Ann. Anatomy-Anatomischer Anzeiger, vol. 238, Nov. 2021, Art. no. 151763. 10.1016/j.aanat.2021.151763 34051322

[62] L. Salem, J. Rey, S. P. Roddy, and R. C. Darling, “Duplex utilization of radial artery imaging,” in Noninvasive Vascular Diagnosis, London, U.K.: Springer, 2013, pp. 379–385.

[63] T. Katsuura, S. Izumi, M. Yoshimoto, H. Kawaguchi, S. Yoshimoto, and T. Sekitani, “Wearable pulse wave velocity sensor using flexible piezoelectric film array,” in Proc. IEEE Biomed. Circuits Syst. Conf. (BioCAS), Oct. 2017, pp. 1–4.

[64] International Organization for Standardization (ISO), Noninvasive Sphygmomanometers—Part 2-61: Clinical Investigation of Intermittent Automated Measurement Type, Standard ISO 81060-2:2018, 2018.

[65] Q. Wang, Y. Cui, N. Lin, and S. Pang, “Correlation of cardiomyocyte apoptosis with duration of hypertension, severity of hypertension and caspase-3 expression in hypertensive rats,” Experim. Therapeutic Med., vol. 17, pp. 2741–2745, Feb. 2019. 10.3892/etm.2019.7249 PMC6425263 30906464

[66] K. L. Christensen, M. J. Mulvany, and L.T. Jespersen, “Can mean arterial pressure be estimated from measurements of systolic and diastolic blood pressure, and vice versa?” J. Hypertension, vol. 8, no. 4, pp. 321–326, Apr. 1990. 10.1097/00004872-199004000-00005 2160488

[67] E. Sidhu, M. Yoshida, V.Z. Groza, H. R. Dajani, and M. Bolic, “Performance analysis of oscillometric blood pressure estimation techniques in cardiac patients,” in Proc. IEEE Int. Symp. Med. Meas. Appl. (MeMeA), Jun. 2019, pp. 1–6.

[68] T.-W. Wang, W.-X. Chen, H.-W. Chu, and S.-F. Lin, “Single-channel bioimpedance measurement for wearable continuous blood pressure monitoring,” IEEE Trans. Instrum. Meas., vol. 70, pp. 1–9, 2021.33776080

[69] A. M. Dart and B. A. Kingwell, “Pulse pressure—A review of mechanisms and clinical relevance,” J. Amer. College Cardiology, vol. 37, no. 4, pp. 975–984, Mar. 2001. 10.1016/s0735-1097(01)01108-1 11263624

