# Supplementary material for: Assessment of a Calibration-Free Method of Cuffless Blood Pressure Measurement: A Pilot Study
Source: IEEE J Transl Eng Health Med. 2022 Sep 26;11:318–29. doi: 10.1109/JTEHM.2022.3209754 (PMC10756135; doi:10.1109/JTEHM.2022.3209754)
Supplement: Supplementary materials [file supp1-3209754.doc]

# Supplementary materials

Fig. S1 In supplementary to Fig.6a, amplification curves measured in each lightbulb are all shown in an array format.

| (a) |  |
| --- | --- |
| (b) |  |

Fig. S2. In supplementary result to TABLE III, fluorescence signals measured from each lightbulb are all shown in an array format. Pathogen nucleic acids with valid amplification curves in replicated qPCR lightbulbs were embraced in red solid-line boxes; whereas, these for three quality controls were embraced in dashed-line boxes. (a) Flu A, Flu A/H1, Flu A/H3, Flu B and *CP* were correctly detected. The detection was robust in all of the replicated lightbulbs. Lightbulbs for quality controls (*SUC1*, *GAPDH* and qPCR control) were also included. (b) Result of a negative control sample. The multiplexed pathogen detections were performed by the 23-plex cartridge of AAMST.
